# The IUPHAR/BPS Guide to PHARMACOLOGY: an expert-driven knowledgebase of drug targets and their ligands

**DOI:** 10.1093/nar/gkt1143

**Published:** 2013-11-14

**Authors:** Adam J. Pawson, Joanna L. Sharman, Helen E. Benson, Elena Faccenda, Stephen P.H. Alexander, O. Peter Buneman, Anthony P. Davenport, John C. McGrath, John A. Peters, Christopher Southan, Michael Spedding, Wenyuan Yu, Anthony J. Harmar, NC-IUPHAR

**Affiliations:** ^1^The University/BHF Centre for Cardiovascular Science, The Queen’s Medical Research Institute, University of Edinburgh, Edinburgh EH16 4TJ, UK, ^2^School of Biomedical Sciences, Life Sciences E Floor, University of Nottingham Medical School, Queen's Medical Centre, Nottingham NG7 2UH, UK, ^3^Laboratory for Foundations of Computer Science, School of Informatics, 10 Crichton Street, University of Edinburgh, Edinburgh EH8 9AB, UK, ^4^Clinical Pharmacology Unit, Level 6, Centre for Clinical Investigation, Box 110, Addenbrooke’s Hospital, University of Cambridge, Cambridge CB2 0QQ, UK, ^5^School of Life Sciences, University of Glasgow, Glasgow G12 8QQ, UK, ^6^Neuroscience Division, Medical Education Institute, Ninewells Hospital and Medical School, University of Dundee, Dundee DD1 9SY, UK and ^7^Spedding Research Solutions SARL, 6 Rue Ampere, Le Vésinet 78110, France

## Abstract

The International Union of Basic and Clinical Pharmacology/British Pharmacological Society (IUPHAR/BPS) Guide to PHARMACOLOGY (http://www.guidetopharmacology.org) is a new open access resource providing pharmacological, chemical, genetic, functional and pathophysiological data on the targets of approved and experimental drugs. Created under the auspices of the IUPHAR and the BPS, the portal provides concise, peer-reviewed overviews of the key properties of a wide range of established and potential drug targets, with in-depth information for a subset of important targets. The resource is the result of curation and integration of data from the IUPHAR Database (IUPHAR-DB) and the published BPS ‘Guide to Receptors and Channels’ (GRAC) compendium. The data are derived from a global network of expert contributors, and the information is extensively linked to relevant databases, including ChEMBL, DrugBank, Ensembl, PubChem, UniProt and PubMed. Each of the ∼6000 small molecule and peptide ligands is annotated with manually curated 2D chemical structures or amino acid sequences, nomenclature and database links. Future expansion of the resource will complete the coverage of all the targets of currently approved drugs and future candidate targets, alongside educational resources to guide scientists and students in pharmacological principles and techniques.

## INTRODUCTION

Online resources have become indispensable tools for pharmacology and drug discovery, in common with other disciplines in the biomedical sciences. Databases such as ChEMBL (1) and PubChem (2) provide extensive information on the bioactivity and chemical structures of approved and experimental drugs and their interaction with targets, either manually curated from the medicinal chemistry literature (ChEMBL) or uploaded by depositors (PubChem). To complement these large-scale resources, there is a need for an in-depth, expert-curated overview of the key targets and ligands, to foster basic and clinical research and innovative drug discovery, and to educate the next generation of researchers. The International Union of Basic and Clinical Pharmacology/British Pharmacological Society (IUPHAR/BPS) Guide to PHARMACOLOGY portal (http://www.guidetopharmacology.org) is being developed to assist research in pharmacology, drug discovery and chemical biology in academia and industry, by providing: (i) an authoritative synopsis of the complete landscape of current and research drug targets; (ii) an accurate source of information on the basic science underlying drug action; (iii) guidance to researchers in selecting appropriate compounds for *in vitro* and *in vivo* experiments, including commercially available pharmacological tools for each target; and (iv) an integrated educational resource for researchers, students and the interested public.

The Guide to PHARMACOLOGY portal has been online since December 2011. The current release of the database (October 2013) integrates two well-established sources. The first of these is the IUPHAR Database [IUPHAR-DB: (3)], which provides in-depth, integrative views of the pharmacology, genetics, functions and pathophysiology of important target families, including G protein-coupled receptors (GPCRs), ion channels and nuclear hormone receptors (NHRs). The second is the BPS ‘Guide to Receptors and Channels’ [GRAC: (4)], a compendium, previously published in print, providing concise overviews of the key properties of a wider range of targets than those covered in IUPHAR-DB, together with their endogenous ligands, experimental drugs, radiolabelled ligands and probe compounds, with recommended reading lists for newcomers to each field.

Management and peer review of the new resource is the responsibility of the IUPHAR Committee on Receptor Nomenclature and Drug Classification (NC-IUPHAR), which acts as the scientific advisory and editorial board. The organization has an international network of over 700 expert volunteers organized into ∼60 subcommittees dealing with individual target families. The subcommittee members contribute expertize in several ways, including identifying the key pharmacological properties of each target, along with quantitative activity data from the research literature. NC-IUPHAR also directly supports the Guide to PHARMACOLOGY through its work in monitoring ‘deorphanization’ of receptors (i.e. identifying new endogenous ligands), revising receptor nomenclature in collaboration with HUGO Gene Nomenclature Committee (HGNC) database (5–7), liaising with journals, and developing standards and terminology in quantitative pharmacology (8–10).

**Figure 1.**
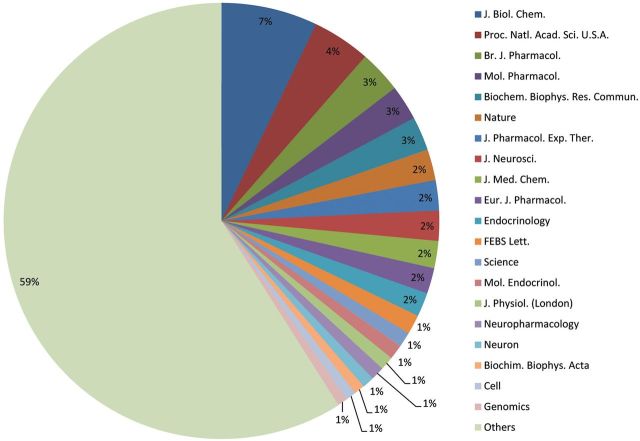
Breakdown of scientific journals cited in the resource. The chart shows the top 20 most cited journals in the resource, and the contribution of each journal as a percentage of the total.

The primary sources of data in the Guide to PHARMACOLOGY are distinct from the medicinal chemistry and natural product literature extracted by ChEMBL. Our focus is on data and contextual information relevant to the preclinical phases of drug discovery and includes extensive quantitative and chemical information manually curated from the primary research literature, predominantly from the leading non-specialist scientific journals and widely read specialist journals (Figure 1).

## CONTENT AND DATA CURATION

The current version of the database includes pharmacologically relevant data and information on 2485 human targets including GPCRs, ion channels, NHRs, catalytic (enzyme linked) receptors, transporters and enzymes (including all protein kinases) (Table 1). Also included, is information on the genetics, emerging pharmacology, functions and pathophysiology of 130 orphan GPCRs (7).

**Table 1.** Database statistics

Target class	Number of targets
7TM receptors	400
GPCRs including orphans	394
Orphan GPCRs	130
Other 7TM proteins	6
Nuclear hormone receptors	48
Catalytic receptors	223
Ligand-gated ion channels	84
Voltage-gated ion channels	142
Other ion channels	49
Enzymes	1008
Transporters	503
Other protein targets	28
Total number of targets	2485

Chemical class	Number of ligands
Synthetic organics	3504
Metabolites	550
Endogenous peptides	687
Other peptides including synthetic peptides	1089
Natural products	161
Antibodies	10
Inorganics	55
Others	8
Approved drugs	559
Withdrawn drugs	11
Drugs with INNs	857
Radioactive ligands	550
Total number of ligands	6064
Number of synonyms	51189
Number of binding constants	41076
Number of references	21774

Presently, the resource describes the interactions between target proteins and 6064 distinct ligand entities (Table 1). Ligands are listed against targets by their action (e.g. activator, inhibitor), and also classified according to substance types and their status as approved drugs. Classes include metabolites (a general category for all biogenic, non-peptide, organic molecules including lipids, hormones and neurotransmitters), synthetic organic chemicals (e.g. small molecule drugs), natural products, mammalian endogenous peptides, synthetic and other peptides including toxins from non-mammalian organisms, antibodies, inorganic substances and other, not readily classifiable compounds.

**Figure 2.**
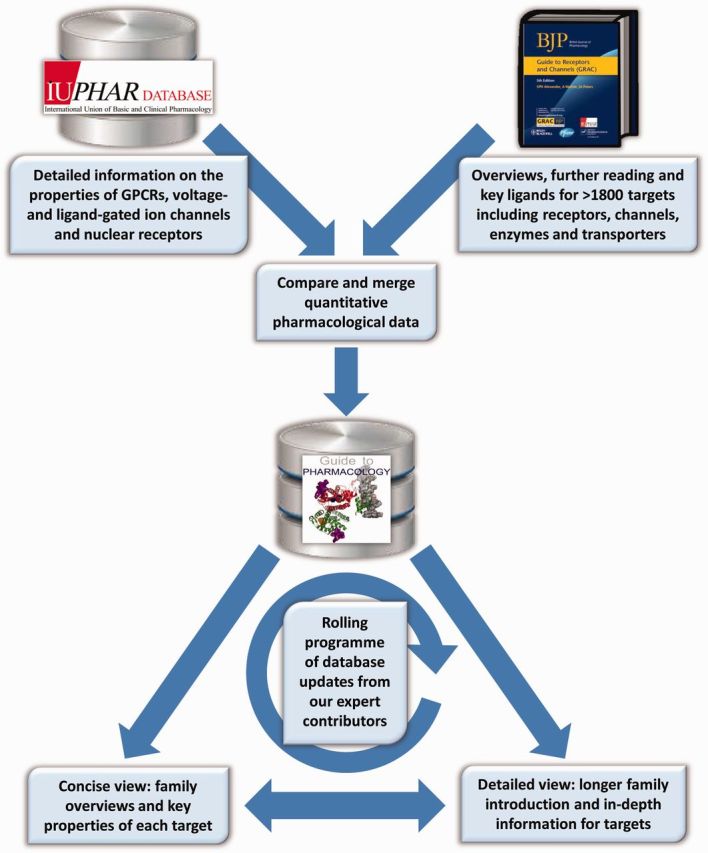
The Guide to PHARMACOLOGY curation process and organizational chart.

**Figure 3.**
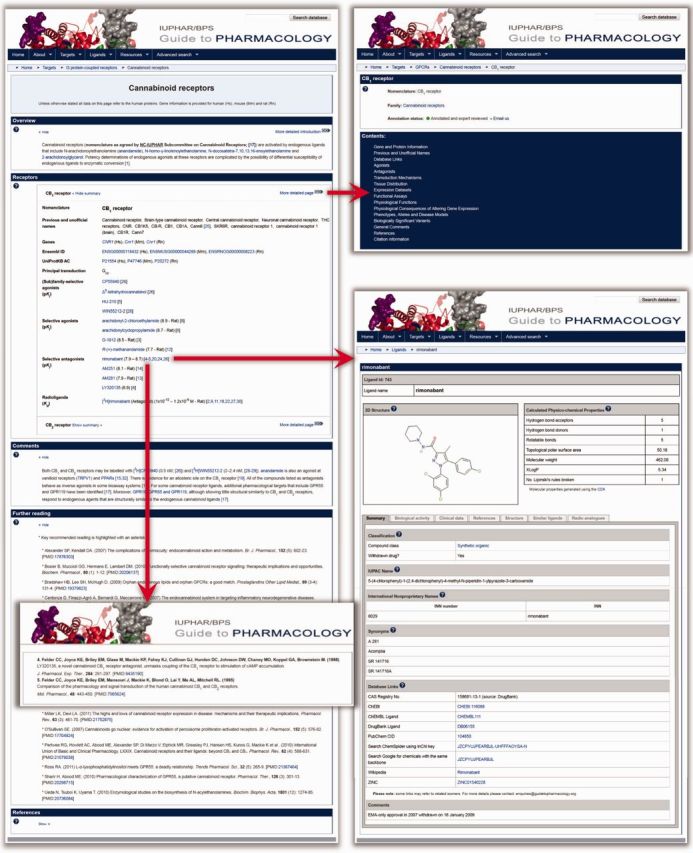
Screenshot of the Cannabinoid receptor family page in the Guide to PHARMACOLOGY, with overlaying screenshots of a typical ligand page and reference page with link-out to PubMed. Also shown is a link to the ‘More detailed page’ of the CB_1_ receptor with a screenshot of the top section of the target page showing the ‘Contents’ table listing the types of information available for this target.

The new database was constructed by integrating data from IUPHAR-DB (3) and the published GRAC compendium (4). An overview of the curation process is depicted as an organizational flow chart in Figure 2. New information was added to the existing relational database behind IUPHAR-DB and new webpages were created to display the integrated information. For each new target, information on human, mouse and rat genes and proteins, including gene symbol, full name, location, gene ID, UniProt and Ensembl IDs was manually curated from HGNC (5), the Mouse Genome Database (MGD) at Mouse Genome Informatics (MGI) (11), the Rat Genome Database (RGD) (12), UniProt (13) and Ensembl (14), respectively. In addition, ‘Other names’, target-specific fields such as ‘Principal transduction’, text from the ‘Overview’ and ‘Comments’ sections and reference citations (downloaded from PubMed; http://www.ncbi.nlm.nih.gov/pubmed) were captured from GRAC and uploaded into the database against a unique Object ID. For targets present in both IUPHAR-DB and GRAC, entries were cross-checked and merged. A representative target family page is shown in Figure 3.

For the integration exercise, all ligands listed in GRAC were first checked against IUPHAR-DB using name-, synonym- and structure-based comparisons. For over 1000 ligands, there was an existing IUPHAR-DB entry that matched. The remaining new ligands (∼1900) were curated using the workflow already established for the population of IUPHAR-DB with ligand structures (15). An overview of the process is outlined below.

Interrogation of multiple databases and direct literature checks captured the correct structural information, nomenclature and target mapping for each ligand. All small molecules were resolved against a PubChem Compound Identifier (CID) as a primary molecular identifier and representative chemical structure (2). Each ligand was then uploaded into the resource with a unique ID. The quantitative pharmacological activity data of each ligand was captured from GRAC and uploaded.

Ligands have individual pages (Figure 3) providing 2D chemical structures or peptide sequences, calculated physico-chemical properties, classification and approval status for human clinical use, the International Union of Pure and Applied Chemistry (IUPAC) name and other names used as synonyms. International Nonproprietary Names (INNs) are also currently provided for 730 compounds. INNs are the official non-proprietary or generic names given to pharmaceutical substances, as designated by the World Health Organization (WHO; http://www.who.int/medicines/services/inn/en/). For small molecules, simplified molecular input line entry specification (SMILES), the IUPAC International Chemical Identifiers (InChI string and InChIKey) and Chemical Abstracts Service (CAS) registry numbers (http://www.cas.org/index.html) are provided. Peptides are specified by one- and three-letter amino acid sequences, any post-translational modifications and details of their protein precursors. Links are provided to corresponding entries in relevant bioactivity and chemistry resources including BindingDB (16), Chemical Entities of Biological Interest (ChEBI) (17), ChEMBL (1), ChemSpider (18), DrugBank (19), Human Metabolome Database (HMDB) (20), PharmGKB (21), RCSB Protein Data Bank (22), UniProt (13) and ZINC (23). Ligand pages also display a list of structurally similar ligands and a summary of all biological activity data for each compound across all the targets.

The ligand page includes an option to display the results for InChIKey searching in Google, the utility of which has recently been described (24). While the entire Key is used for exact-match searches of ChemSpider, the Google search uses just the inner ‘layer’ of 14 characters approximating to the basic molecular connectivity. It will thus retrieve all related entries with isomeric differences encoded in the outer layer of the Key. The results, typically returned in <0.5 s with very high specificity, are the matches from over 50 million InChIKeys cached by Google from a wide range of databases and web resources.

## IMPLEMENTATION

The data are held in a PostgreSQL relational database (http://www.postgresql.org), with the exception of ligand structures and physico-chemical properties, which are stored in an Oracle database (Oracle Corporation, Redwood Shores, CA, USA). Curators use custom-built Java (Oracle Corporation, Redwood Shores, CA, USA) software to enter and edit data. The public web interface is implemented using HTML, CSS and JavaScript components generated dynamically on the server side by Java servlets and Java Server Pages. The web application runs in the Apache Tomcat servlet container (http://tomcat.apache.org/) on a Linux platform. Ligand structure-based searching is implemented with the Pinpoint chemical cartridge (Dotmatics Limited, Bishops Stortford, UK) and chemical structure editing capability is provided by the MarvinSketch chemical editor (ChemAxon Limited, Budapest, Hungary). Ligand chemical structure formats and identifiers were generated using the Open Babel software (25). IUPAC names were generated using JChem for Excel (ChemAxon Limited, Budapest, Hungary) and physico-chemical properties were generated using the Chemistry Development Kit (26). Ligand images were created using the NCI/CADD Chemical Identifier Resolver from the National Cancer Institute (http://cactus.nci.nih.gov/chemical/structure). Small molecule ligands with similar structures were clustered using Pipeline Pilot (Accelrys, San Diego, CA, USA) and peptides with similar sequences were clustered using h-cd-hit, part of the CD-HIT Suite (27).

## WEB INTERFACE

Users can access ‘Target’ and ‘Ligand’ lists and search tools directly from the portal homepage, as well as from the navigation bar at the top of every subsequent webpage. Each class of target (e.g. transporters, enzymes) is listed according to protein family (e.g. ATP-binding cassette family, amino acid hydroxylases). The portal is designed to provide users with access to two views of pharmacologically relevant data on the targets in the database. The organization and content of these two complementary views is described below:

1. Users are initially presented with concise, searchable overviews of the properties of each family of targets. Data on all members of a target family, or subfamily, are presented on a single webpage (Figure 3). The page for each target family includes a brief overview of the properties of the target group. Details are provided on approved nomenclature (where applicable, approved by NC-IUPHAR) and synonyms, human, mouse and rat gene names and links to the HGNC, MGD, RGD, Ensembl and UniProt databases. Quantitative data are provided on recommended ligands classified by their mode of action (e.g. agonists, antagonists, substrates, inhibitors and radiolabelled ligands) and other information specific to the class of target (e.g. the signal transduction mechanisms used by GPCRs, or the biophysical properties of ion channels). Overall, the data focus on human proteins and include only key pharmacological agents, chosen because they are likely to be the most useful in the laboratory (i.e. they are selective and available by donation, or from commercial sources). A list of review articles recommended as further reading, key references and additional commentary (highlighting, for example, where species differences, or ligand metabolism, are potential confounding factors) are also provided. These pages are designed to serve as an introduction to a family of targets and are a useful entry point into the literature for newcomers to a particular field.
2. From the family overview pages, users can then navigate (*via* the ‘More detailed page’ links, see Figure 3) to database pages with more in-depth information for a subset of important targets, providing expanded views of the pharmacology, genetics, functions and pathophysiology. These include a longer introduction to the family and separate pages providing a comprehensive description of each target and its function, with information on protein structure, ligand interactions, signalling mechanisms, tissue distribution, functional assays and biologically important variants (e.g. single nucleotide polymorphisms and splice variants). Reported ligand interactions may include endogenous ligands, current and historical licensed and experimental drugs, and available radiolabelled ligands, along with information on their actions (e.g. agonist, allosteric modulator, inhibitor) and quantitative data, where possible from multiple literature sources. Comparative data for mouse and rat species are also listed. In addition, the phenotypes resulting from altered gene expression (e.g. in genetically altered animals or in human genetic disorders) are described. An extensive set of links is provided to other resources including protein, gene, structure, disease and drug target databases. Family-specific information and database links are also provided, such as Enzyme Commission (EC) numbers and links to the KEGG BRITE hierarchy describing enzymatic reactions (28). For further details on the types of information that are provided in the detailed view see previous publications (3,15,29).

All literature citations in both views are linked to PubMed, and all ligand entries are linked to individual ligand pages providing additional information (as described in the section on ‘CONTENT AND DATA CURATION’ above).

The interface includes a simple search box where users can enter keywords such as ligand or target names, and advanced search tools which allow searches by specific database field, database identifier (e.g. Ensembl ID), chemical identifier (e.g. standard InChIKey, CAS registry number) or PubMed identifier. Chemical structure searches can also be performed by providing a structure in SMILES format, or drawing a chemical structure using the structure editor. The search tool can perform exact match, substructure, similarity and SMARTS-pattern searches (http://www.daylight.com/dayhtml/doc/theory/theory.smarts.html). The chemical structure editor is also accessible from ligand pages; clicking on the ligand image loads the structure into the editor where it can be modified and used to search the database. Search results indicate which database fields matched the query term, and links are provided to the relevant database entries.

Extensive help pages and a tutorial on how to use the resource are also provided. The help page can be accessed *via* linked icons within database fields as well as from the navigation menu and home page. The help page includes definitions of terms used to describe the data displayed on the site, in addition to providing a detailed guide to using the various search functions.

## COMPARISON WITH OTHER RESOURCES

There are other databases that have a degree of conceptual and content overlap with the Guide to PHARMACOLOGY, some of which are included in this issue. Of these, ChEMBL, DrugBank and Therapeutic Target Database (TTD) (30) are the closest. However, the Guide to PHARMACOLOGY differs from these resources in a number of important ways. Firstly, we restrict the range of protein targets and ligands to those most relevant to therapeutics and drug discovery, chosen with the exercise of curatorial judgement and backed by our network of experts, with a focus on the quality and depth of annotation. Secondly, this is subject to review and quality control, not only by our international expert committee members operating as a *de facto* network of ‘super-curators’, but also *via* user feedback. Thirdly, we curate activity data for research compounds from primary literature sources, including posters and patents, rather than from review articles, with a focus on the interactions of each compound with its data-supported primary target (e.g. Angiotensin-converting enzyme (*ACE*) for captopril). Fourthly, the data can be annotated with free-text comments that would otherwise not easily fit into database schema. These include information on alternative isomers and salt forms. An example here are the eight approved drug–prodrug pairs for ACE inhibitors that present a particular curatorial challenge (e.g. see http://www.guidetopharmacology.org/GRAC/LigandDisplayForward?ligandId=6352). These 16 structures are not both explicitly linked and activity-mapped in other databases.

Another example that illustrates the differences between the three databases is atorvastatin. In the Guide to PHARMACOLOGY (http://www.guidetopharmacology.org/GRAC/LigandDisplayForward?tab=biology&ligandId=2949), there are three activity mappings between this ligand and the primary drug target hydroxymethylglutaryl-CoA reductase (*HMGCR*) with both a *K*_i_ (14 nM) and an IC_50_ for human (8 nM), together with an IC_50_ for rat (1.16 nM). The equivalent DrugBank entry (DB01076) is mapped to 3 targets, 11 enzymes and 9 transporters, but these include associations from the literature that are not all supported by directly measured molecular interactions. The ChEMBL entry (CHEMBL1487) is assay-mapped to 117 proteins and lists 217 IC_50_ values, including proteins in the DRUGMATRIX screen and some antimalarial parasite results. There are four IC_50_ values for the rat and three for the human enzyme. In comparison, the two literature references for atorvastatin in TTD are not the same as from the other three sources. Mapping differences between ChEMBL, DrugBank and TTD have previously been explored in detail (24,31), but the overall picture between these and the Guide to PHARMACOLOGY is one of complementarity. We thus suggest that pharmacologically oriented users might find the curatorially selected set of stringent activity mappings in the Guide to PHARMACOLOGY a simpler entry point (indeed we designed it with this in mind) but we provide extensive linking to the other high-value resources.

## SUMMARY AND FUTURE DIRECTIONS

Our goal is to complete a stringently curated direct mapping (where the primary literature data permits) between chemical structures and their primary molecular targets, initially for targets of approved drugs, but extending this to clinical and research targets. Published listings and the exact definitions for these categories vary widely, but indicate a range of ∼200–300 for the former and ∼500–1000 for the latter (32–36). Possible reasons for disparities in these numbers are indicated in database comparison reports (24,31). We are also in the process of updating our ligand structure submissions to PubChem, facilitating UniProt cross references for their targets and reviewing new information sources for possible inclusion.

The creation of the new portal reflects our intention to develop the resource into a comprehensive online guide, which will include educational resources, and to produce a ‘Concise Guide to PHARMACOLOGY’, to be published in PDF format at two yearly intervals, as a supplement to the *British Journal of Pharmacology*. The ‘Concise Guide to PHARMACOLOGY’, which replaces GRAC, will be a biennial snapshot of succinct overviews of the properties of each target family, intended to be a quick desktop reference guide. Additionally, this will provide a permanent record (DOI: digital object identifier) that will survive database updates and therefore allow the precise context of the database to be understood at any time in the future (37).

Since the Guide to PHARMACOLOGY portal now integrates data from the printed GRAC compendium and IUPHAR-DB, we are planning a phased retirement of IUPHAR-DB. The current URL (http://www.iuphar-db.org) will remain active, with appropriate notices directing users to the Guide to PHARMACOLOGY portal.

## DATA ACCESS

The Guide to PHARMACOLOGY is available online at http://www.guidetopharmacology.org. The website includes downloadable files containing current receptor and channel lists, NC-IUPHAR nomenclature, synonyms, genetic information, HGNC gene nomenclature and identifiers, and other database accessions. Other file formats are available by emailing enquiries@guidetopharmacology.org. Information on linking to Guide to PHARMACOLOGY pages is provided at http://www.guidetopharmacology.org/linking.jsp. To further facilitate external programmatic and user access to the database, we are developing an application programming interface (API) and Web services. This will allow our content to be exploited in new integration initiatives such as Open PHACTS (38), of which we are already an associate member. The database is licensed under the Open Data Commons Open Database License (ODbL) (http://opendatacommons.org/licenses/odbl/), and its contents are licensed under the Creative Commons Attribution-ShareAlike 3.0 Unported license (http://creativecommons.org/licenses/by-sa/3.0/).

## CITING THE RESOURCE

For a general citation of the resource we recommend citing this article. Citation formats for specific target pages are provided on the website.

## FUNDING

International Union of Basic and Clinical Pharmacology; British Pharmacological Society; Wellcome Trust [099156/Z/12/Z]. Funding for open access charge: Wellcome Trust.

*Conflict of interest statement*. None declared.
